# Effects of Barefoot and Shod on the *In Vivo* Kinematics of Medial Longitudinal Arch During Running Based on a High-Speed Dual Fluoroscopic Imaging System

**DOI:** 10.3389/fbioe.2022.917675

**Published:** 2022-06-28

**Authors:** Wanyan Su, Shen Zhang, Dongqiang Ye, Xiaole Sun, Xini Zhang, Weijie Fu

**Affiliations:** ^1^ School of Kinesiology, Shanghai University of Sport, Shanghai, China; ^2^ School of Physical Education and Training, Shanghai University of Sport, Shanghai, China; ^3^ Key Laboratory of Exercise and Health Sciences of Ministry of Education, Shanghai University of Sport, Shanghai, China; ^4^ Shanghai Frontiers Science Research Base of Exercise and Metabolic Health, Shanghai University of Sport, Shanghai, China

**Keywords:** dual fluoroscopic imaging system, barefoot, shod, medial longitudinal arch, *in vivo* kinematics

## Abstract

Shoes affect the biomechanical properties of the medial longitudinal arch (MLA) and further influence the foot’s overall function. Most previous studies on the MLA were based on traditional skin-marker motion capture, and the observation of real foot motion inside the shoes is difficult. Thus, the effect of shoe parameters on the natural MLA movement during running remains in question. Therefore, this study aimed to investigate the differences in the MLA’s kinematics between shod and barefoot running by using a high-speed dual fluoroscopic imaging system (DFIS). Fifteen healthy habitual rearfoot runners were recruited. All participants ran at a speed of 3 m/s ± 5% along with an elevated runway in barefoot and shod conditions. High-speed DFIS was used to acquire the radiographic images of MLA movements in the whole stance phase, and the kinematics of the MLA were calculated. Paired sample t-tests were used to compare the kinematic characteristics of the MLA during the stance phase between shod and barefoot conditions. Compared with barefoot, shoe-wearing showed significant changes (*p* < 0.05) as follows: 1) the first metatarsal moved with less lateral direction at 80%, less anterior translation at 20%, and less superiority at 10–70% of the stance phase; 2) the first metatarsal moved with less inversion amounting to 20–60%, less dorsiflexion at 0–10% of the stance phase; 3) the inversion/eversion range of motion (ROM) of the first metatarsal relative to calcaneus was reduced; 4) the MLA angles at 0–70% of the stance phase were reduced; 5) the maximum MLA angle and MLA angle ROM were reduced in the shod condition. Based on high-speed DFIS, the above results indicated that shoe-wearing limited the movement of MLA, especially reducing the MLA angles, suggesting that shoes restricted the compression and recoil of the MLA, which further affected the spring-like function of the MLA.

## 1 Introduction

Given the increased investigation and analysis of running biomechanics, the function and material of running shoes, such as cushioned shoes, have been developed (Hannigan and Pollard, 2020; Fu et al., 2021; Li et al., 2022). Running shoes were designed to attenuate impact forces *via* the viscose-elastic midsole (Nigg et al., 2003; Bishop et al., 2006), which can be compressed and rebounded during the loading and unloading periods of the running cycle (Baltich et al., 2015), thus effectively avoiding lower limb injuries caused by repeated high-intensity impact loads (Nigg et al., 2003; Sun et al., 2020; Kakouris et al., 2021).

During running, the foot is an important structure connecting the ground to the body, adapting to the running surface, affecting energy absorption and transfer (Welte et al., 2018). The medial longitudinal arch (MLA) allows the foot to function in a spring-like manner which is crucial in energy storage and release (Lynn et al., 2012). During the early and mid-stance phases, MLA compresses and lowers its height, whereas mechanical energy is absorbed and stored in the elastic structures of the MLA (Ker et al., 1987). During propulsion, the dorsiflexion (DF) of the first metatarsophalangeal joint tightened the plantar fascia (Hicks, 1954; Bolgla and Malone, 2004), thus, the MLA is shortened, and height is increased, accompanied by the elastic rebound of soft tissue and release of 17% mechanical energy (Ker et al., 1987; Kelly et al., 2015). The deformation of MLA directly affects the arch-spring behavior and the energetics of the foot.

From the perspective of MLA function, wearing shoes limit the DF of the metatarsophalangeal joint before initial contact during gait, it may further restrict the elongation of the MLA during impact, attenuates the magnitude of MLA deformation, and subsequently affect the MLA’s ability to absorb shock (Wegener et al., 2015; Welte et al., 2018). Moreover, the long-term wearing of cushioned shoes leads to over-reliance on the shoes to absorb shock rather than the foot intrinsic muscles and tendons, which may potentially weaken the foot intrinsic muscle and spring-like function of MLA (Lieberman et al., 2010; Davis, 2014).

Knowing the accurate movement of MLA inside a shoe may contribute to further understanding of the influence of shoes on MLA during running. In previous studies, the trajectories of reflective markers with motion capture systems were used to measure MLA movement. However, the relative movement between the marker and the bone may cause soft tissue artifacts (STA), which cannot accurately reflect bone movement (Shultz et al., 2011). Intracortical pins can eliminate the effects of STA, but they are an invasive and infectious procedure for the human body (Arndt et al., 2013). A high-speed dual fluoroscopic imaging system (DFIS), combined with fluoroscopic imaging, medical imaging, and 3D-2D model registration technologies, can transcend the limitations of traditional biomechanical motion capture methods, that capture and quantify the dynamic movement of bones *in vivo* without STA and invasion (Ye et al., 2021). Moreover, a few studies recently published have used DFIS to measure skeletal foot kinematics *in vivo* during walking (Balsdon et al., 2016; Phan et al., 2018; Balsdon et al., 2019; Phan et al., 2019). DFIS has been used to measure the relative movement of the tibiotalar to subtalar joints (Phan et al., 2018) and midtarsal joint locking (Phan et al., 2019) and MLA angle (Balsdon et al., 2016; Balsdon et al., 2019) during walking. Given the above, the application of DFIS in the field of biomechanics provides a new perspective for accurate analysis of the *in vivo* kinematics of the foot. However, in contrast to this study that using high-speed DFIS, previous studies were based on normal DFIS. No studies to date have used high-speed DFIS to quantify the movement of the MLA under shod and barefoot conditions during running. This study based on high-speed DFIS may provide insights into the accurate movement of the MLA and a reference for future assessment of the potential association between abnormal MLA movement and injury.

Thus, this study aimed to investigate the *in vivo* kinematics of the MLA during running in barefoot and shod conditions using high-speed DFIS. We hypothesized that compared with barefoot, wearing shoes limits the six degrees of freedom (6DOF) movement of the MLA.

## 2 Methods

### 2.1 Participants

Fifteen healthy male participants (29.1 ± 6.9 years, 173.0 ± 4.5 cm, 71.7 ± 7.3 kg, and 38.8 ± 16.6 km/week) were included in the study. Sample size estimation by G*power (Version 3.1.9.6, Kiel University, Kiel, Germany) indicated that a minimum sample size of 15 participants was required to achieve a minimum effect size of 0.8 (significance level: 0.05, statistical power: 0.8). The inclusion criteria were habitual rearfoot strike runners and absence of pain and injury in the lower limbs for at least 3 months before enrollment in the study. All participants were right foot dominant (dextropedal). All participants provided informed consent, and the study protocol, including computed tomography (CT) scan and high-speed DFIS, was approved by the Institutional Review Board at Shanghai University of Sport, Shanghai, China (Approval No.102772021RT034).

### 2.2 Computed Tomography Scanning and Three-Dimensional Bone Modeling

All participants underwent CT scanning (SOMATOM, Germany) of their right foot for the creation of the 3D model used in postprocessing. A brace was used to fix the ankle and foot in a neutral position with the participants in the supine position during CT scanning. The main CT scanning parameters were as follows: thickness, 0.6 mm; voltage, 120 kV; current, 140 mA; image matrix, 512 × 512 × 256 pixels. The voxel length, width, and height were set as 0.488, 0.488, and 0.625 mm, respectively. The scanning scale ranged from 10 cm above the ankle joint to the end of the toes (Cao et al., 2019).

**FIGURE 1:**
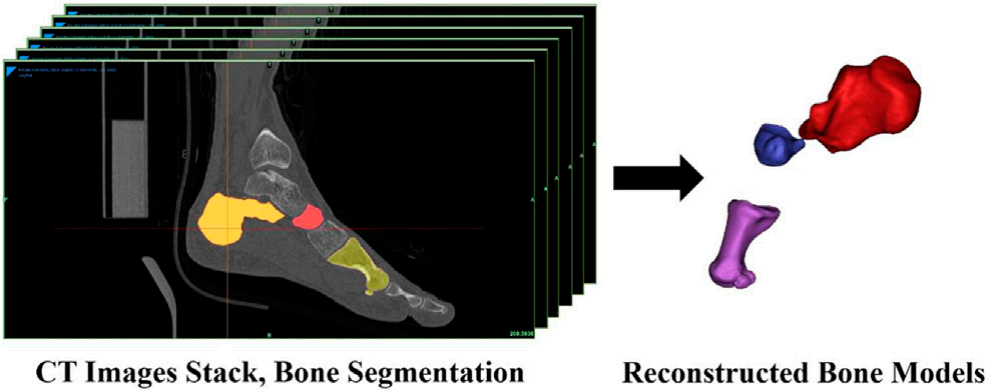
Reconstruction of the first metatarsal, navicular, and calcaneus.

All CT images were subsequently imported into a 3D reconstruction software (v.21.0; Mimics, Materialise, Leuven, Belgium). The models of the first metatarsal, calcaneus, and navicular bones were reconstructed *via* threshold and region growth in Mimics (Figure 1). According to previous studies, we used the first metatarsal, navicular, and calcaneus to quantify the movement of the MLA (Simon et al., 2006; Tome et al., 2006; Bandholm et al., 2008; Balsdon et al., 2016; Welte et al., 2021).

### 2.3 Establishment of a Coordinate System

The three orthonormal axes (xyz) were aligned with the XYZ axes of the global coordinate system at the zero-loading condition on the level surface, and the inertial anatomical coordinate systems were generated from the bone meshes with the origin located at the centroid (Eberly et al., 1991; Negishi et al., 2022). The coordinate system axes were re-labeled such that the x-, y-, and z-axes more closely represented the lateral, anterior, and superior (Welte et al., 2021). We quantified the changes in the positions of the bones base on their origins.

### 2.4 Data Collection

The high-speed DFIS consisted of two pairs of fluorescence emitters that can generate X-rays and two image intensifiers (diameter: 431.8 mm) that can receive and image X-rays. In terms of precision, DFIS demonstrated a bias range of −0.16–0.13 mm and −0.05–0.13°, a precision range of 0.05–0.86 mm and 0.06–0.69°, and an overall dynamic root-mean-squared average error of 0.59 mm and 0.71° in static and dynamic trials (Cross et al., 2017). In this study, the distances between the two fluorescence emitters and between image intensifiers were 132.2 and 128.6 cm, respectively. The two image intensifiers were positioned at 119.6° to one another. The shooting voltage was 60 kV, the current was 63 mA, and the shooting frequency was 100 Hz. The exposure speed was 1/1000 s, and the image resolution was 1024 × 1024 pixels. Due to the limited range of high-speed DFIS acquisition, only right foot was investigated during data collection.

**FIGURE 2:**
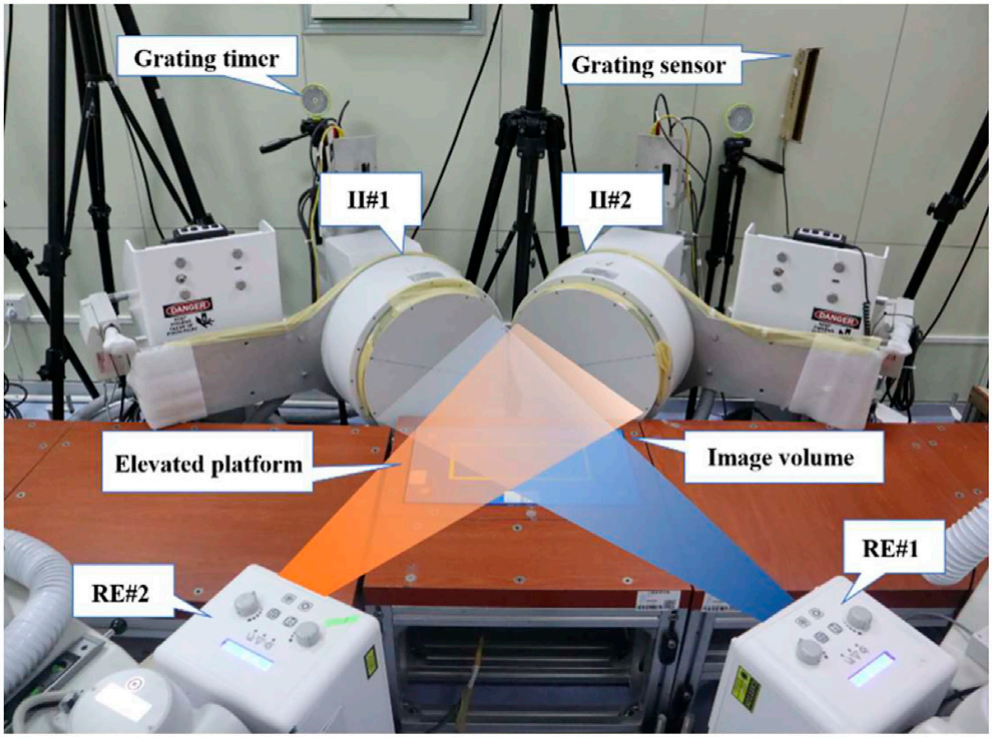
High-speed DFIS set-up. Participants ran on an elevated platform. Image intensifiers (II#1 and II#2) processed images created by X-rays from the radiographic emitters (RE#1 and RE#2).

All participants were provided with a pair of running shoes (traditional footwear, heel-to-toe drop: 6 mm, midsole material: TPU and EVA; without any arch support) before the running experiment. Then, all participants warmed up for 5 min on a treadmill at a speed of 3 m/s. Before initiating the measurement, the participants could practice with and without shoes several times, so that the participants were familiar with the elevated runway under footwear and barefoot conditions for eliminating the effect of practice. During the test, they ran at a speed of 3 m/s ± 5% in barefoot and shod conditions along an elevated runway with their right foot landing within the X-ray volume. Each trial was supervised by the experimenter to ensure that the participants landed naturally. According to the study of Shetty and Bendall (2011), the stance phase was defined as the phase from foot strike to toe-off. The moment that the vertical ground reaction force was larger than 15 N was used to define the “heel-strike” (Welte et al., 2018). The force plate was synchronized with the DFIS *via* a custom synchronized trigger device. During running, the body of the participants blocked the infrared blocking grating sensor and triggered the high-speed DFIS and force plate to collect the data during the stance phase. One successful trial was collected using high-speed DFIS in each condition (Campbell et al., 2016; Welte et al., 2021), guided by the X-ray image quality (Figure 2).

### 2.5 Data Processing

Images obtained with high-speed DFIS were subjected to distortion when X-ray beams were transformed into visible images (Gronenschild, 1999; Tersi and Stagni, 2014). Pincushion and magnetic lens distortions were the two major sources of distortion (Wang and Blackburn, 2000). For calibration, an X-ray image was collected with an un-distorted grid (Brainerd et al., 2010), which consisted of a perforated piece of aluminum plate with a known size and spacing of each hole. An X-ray-specific software (XMALab, Brown University, United States) was used to correct for any changes in the spacing or size of the holes in the perforated grid *via* a distortion-correcting algorithm (Rohr et al., 2001).

**FIGURE 3:**
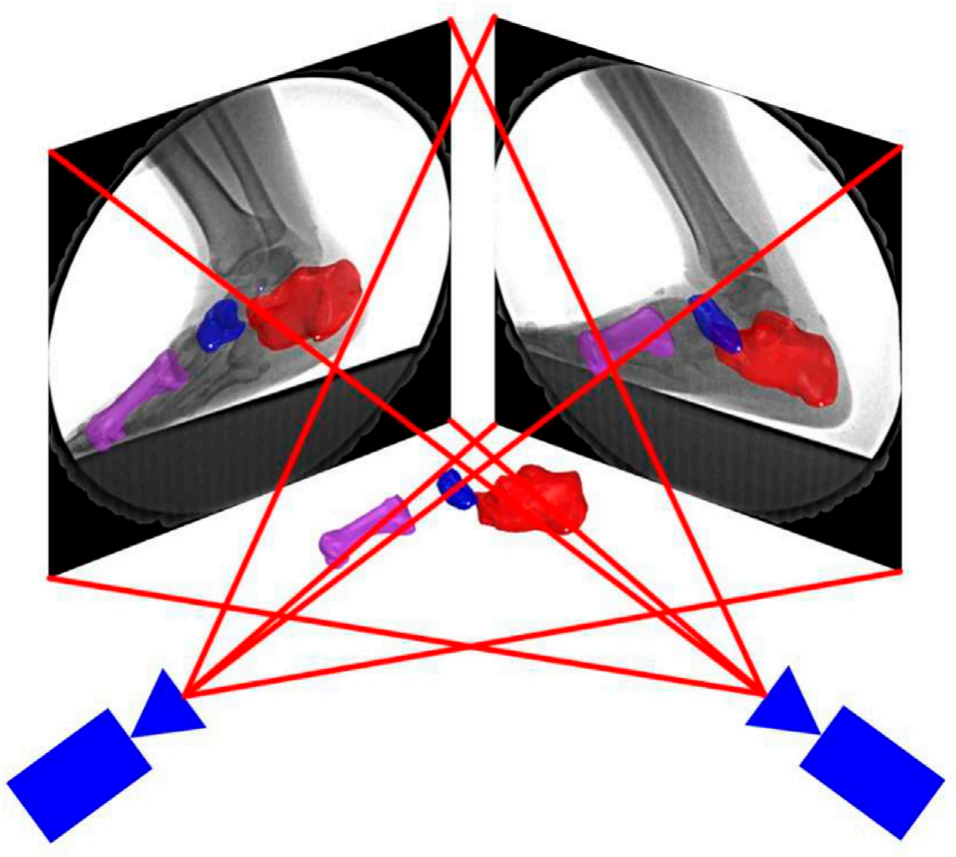
3D–2D registration. Bone positions were adjusted by translation and rotating the bone models in the software until the edge of the bones matched with the radiographic images.

The corrected fluoroscopic images and 3D models of the first metatarsal, navicular, and calcaneus were transferred to the 3D to 2D scientific rotoscoping software (Rhinoceros 6.0, 142 McNeel & Associates, Seattle, United States). The software created a 3D virtual environment of the experiment on a computer (Figure 3).

### 2.6 Parameters

The kinematic results of the MLA were calculated by 3D to 2D registration. The specific indicators included the range of motion (ROM), which was defined as the maximum minus the minimum value among all frames, initial contact value, and peak value. The stance phase was divided into 10 sections with 10% per section. The stance phase was divided into an early stance (0–20%), mid-stance (20–55%), and propulsion (55–85%) (Welte et al., 2021). The kinematics of each counterpart section were compared. The execution time of the joint kinematics during the stance period was standardized *via* an interpolation algorithm in MATLAB (R2018a, MathWorks, Natick, United States).

#### 2.6.1 Definition of Six Degrees of Freedom Kinematics

**FIGURE 4:**
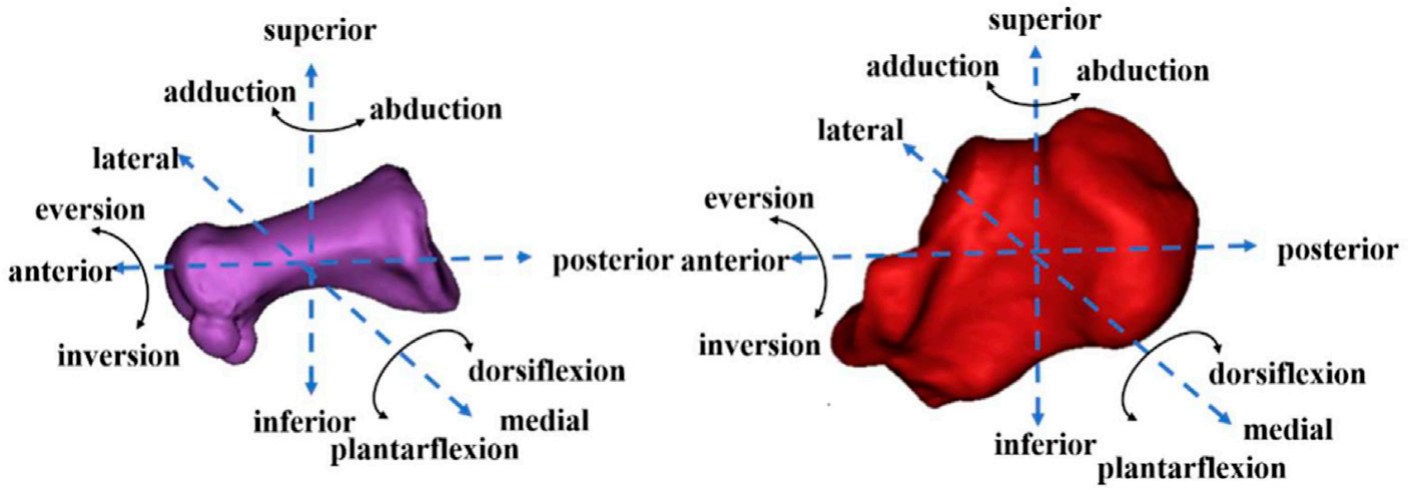
First metatarsal (left) and calcaneus (right) motion diagram.

The 6DOF kinematics of the MLA was defined as the relative movement of the first metatarsal coordinate system with respect to the calcaneus coordinate system (Kelly et al., 2015; Welte et al., 2021). The medial/lateral, anterior/posterior, and superior/inferior directions were aligned with the x-, y-, and z-axes of the coordinate systems, respectively (Wu et al., 2002). Plantar flexion/dorsiflexion (PF/DF), inversion/eversion (IR/ER), and abduction/adduction (AB/AD) were determined as rotations around the medial/lateral, anterior/posterior, and superior/inferior axes, respectively (Wu et al., 2002). The positive values represented anterior translation, lateral translation, superior translation, DF, IR, and AB, and the negative values corresponded to the opposite (Figure 4).

#### 2.6.2 Medial Longitudinal Arch Angle

**FIGURE 5:**
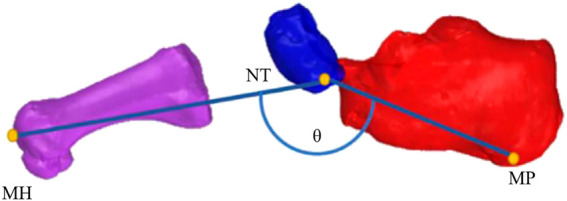
Bones and landmarks defining the MLA angle (θ).

Similar to the MLA measurement used by Tome et al. (2006), landmarks, including the medial process of the calcaneus (MP), navicular tuberosity (NT), and the most distal point on the first metatarsal head (MH), were used to quantify the angle representing the MLA angle. A custom algorithm was used to calculate the spatial vector angles, which represented the MLA angles (θ), using vectors from NT to the MP(NTMP) and the NT to the first MH(NTMH) in the 3D space (Balsdon et al., 2016). A large MLA angle represents a low (flattened) arch height, and a small MLA angle denotes a high (raised) arch height (Figure 5).

### 2.7 Statistics

The mean and standard deviation for each variable were calculated. All variables were normally distributed as indicated by the Shapiro-Wilk test. Paired sample t-test was used to compare the 6DOF data of the MLA and MLA angles under two conditions (SPSS 25.0, IBM, Chicago, United States). The significance level was set as α = 0.05.

## 3 Results

### 3.1 Translation of the First Metatarsal Relative to the Calcaneus

**FIGURE 6:**
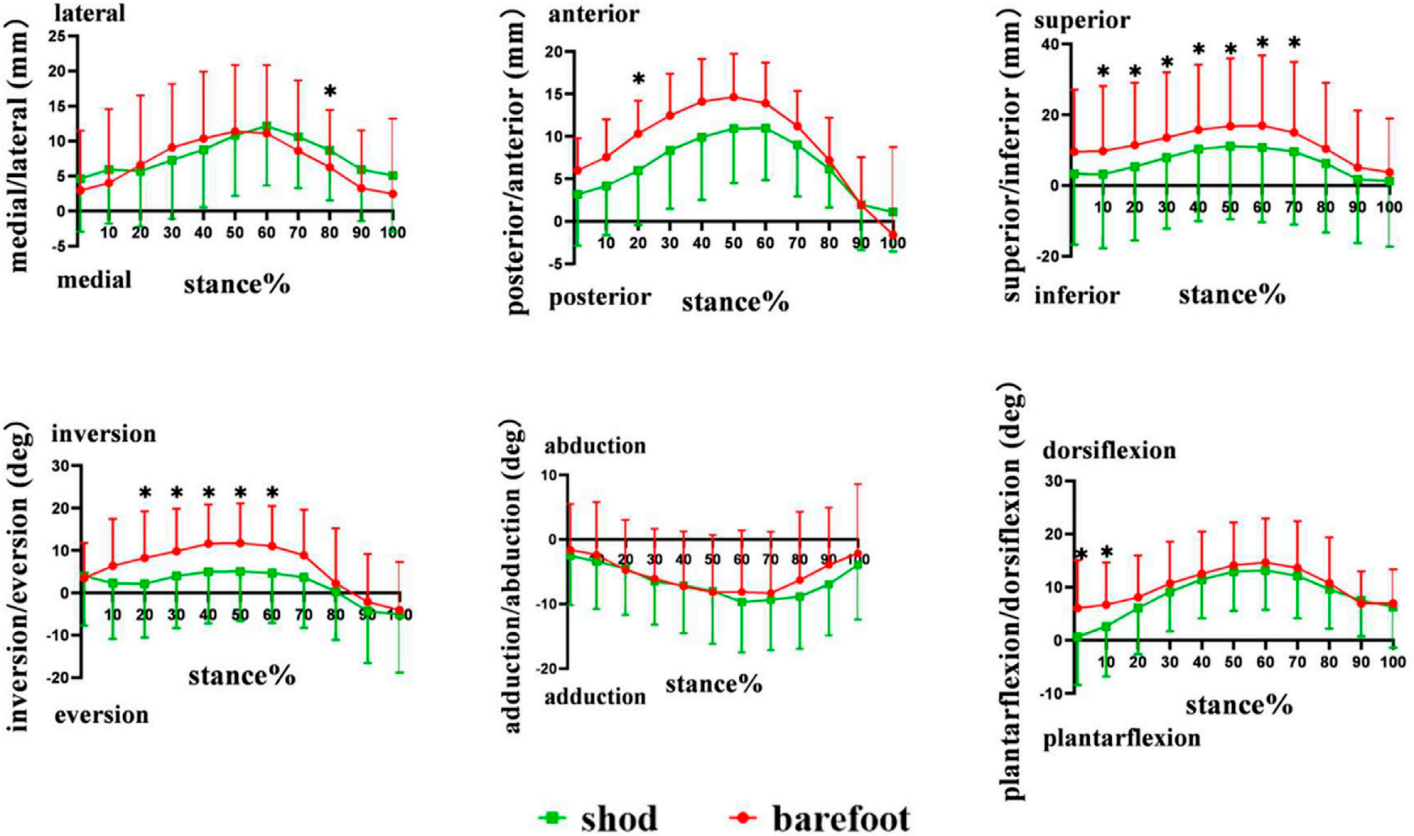
6DOF movement of the first metatarsal relative to the calcaneus during the stance phase. *: significant differences between the shod and barefoot conditions, *p* < 0.05.

Compared with barefoot, the lateral translation at 80% (*p* = 0.046) and the anterior translation at 20% (*p* = 0.029) of the stance phase were smaller in the shod condition. The superior translation at 10% (*p* = 0.010), 20% (*p* = 0.015), 30% (*p* = 0.017), 40% (*p* = 0.027), 50% (*p* = 0.031), 60% (*p* = 0.022) and 70% (*p* = 0.022) of the stance phase was significantly reduced in the shod condition (Figure 6).

**FIGURE 7:**
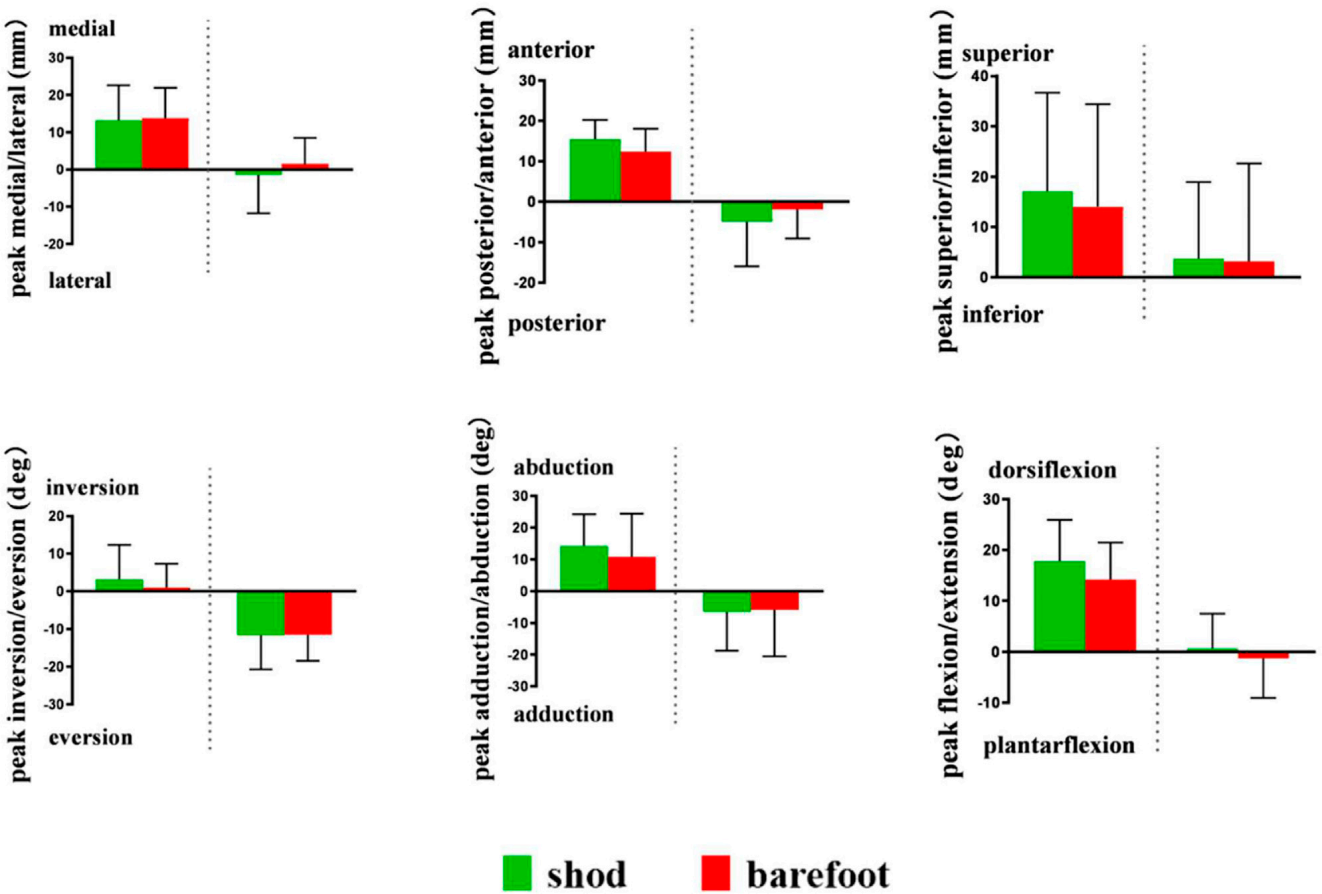
Peak translation and rotation of the first metatarsal relative to calcaneus in shod and barefoot conditions. *: significant differences between the shod and barefoot conditions, *p* < 0.05.

During the stance phase, no significant differences were observed in the initial contact value, peak value, and ROM in the translation of the first metatarsal relative to the calcaneus between shod and barefoot conditions (Figure 7) (Table 1).

**TABLE 1:** Comparison of translation, rotation at initial contact, and ROM of the first metatarsal relative to the calcaneus in shod and barefoot conditions.

	Condition	M/L (mm)	A/P (mm)	S/I (mm)	PF/DF (°)	IR/ER (°)	AB/AD (°)
Initial contact	barefoot	2.96 ± 8.51	5.99 ± 3.79	9.51 ± 17.57	6.05 ± 9.01*	3.48 ± 8.23	−1.61 ± 7.09
	shod	4.65 ± 7.60	3.15 ± 6.01	3.28 ± 20.00	0.63 ± 9.02*	4.03 ± 11.73	−2.51 ± 7.68
ROM	barefoot	14.68 ± 4.35	20.38 ± 11.2	19.31 ± 7.06	17.10 ± 4.90	20.89 ± 9.94*	14.87 ± 3.53
	shod	12.15 ± 2.25	14.73 ± 6.87	15.81 ± 5.49	15.96 ± 4.98	16.45 ± 9.09*	12.54 ± 3.31

*: compared with barefoot, significant differences existed in the shod condition, *p* < 0.05. M/L, medial/lateral translation; A/P, anterior/posterior translation; S/I, superior/inferior translation; PF/DF, plantarflexion/dorsiflexion; IR/ER, inversion/eversion; AB/AD, abduction/adduction; ROM, range of motion; “+“: the first metatarsal medial, anterior, superior translation and DF, IR, and AB; “-“: the first metatarsal lateral, posterior, inferior translation, and PF, ER, and AD.

### 3.2 Rotation of the First Metatarsal Relative to the Calcaneus

Compared with barefoot, the IR angles were smaller at 20% (*p* = 0.033), 30% (*p* = 0.049), 40% (*p* = 0.032), 50% (*p* = 0.021), and 60% (*p* = 0.014), and the DF angles were reduced at initial contact (*p* = 0.002) and 10% (*p* = 0.010) of the stance phase in the shod condition. No significant differences were observed in AB and AD angles during the stance phase (Figure 6).

Compared with barefoot, the IR/ER ROM in the shod condition was significantly smaller (*p* = 0.044). No significant differences were noticed in the peak value of PF/DF, IR/ER, and AD/AB angles between the shod and barefoot conditions (Figure 7) (Table 1).

### 3.3 *In Vivo* Kinematic of the Medial Longitudinal Arch Angles

**FIGURE 8:**
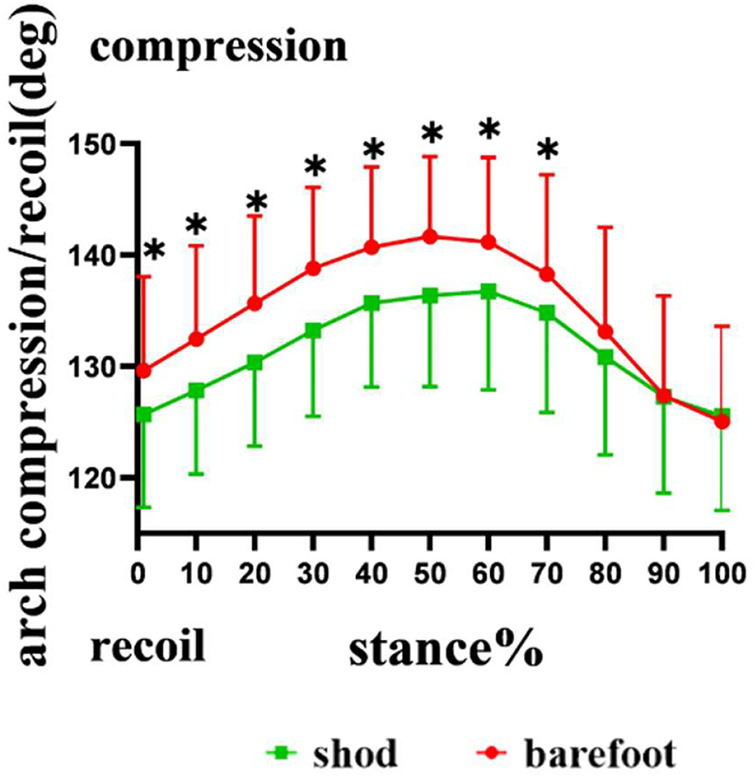
MLA compression and recoil during the stance phase. *: significant differences existed between the shod and barefoot conditions, *p* < 0.05.

**FIGURE 9:**
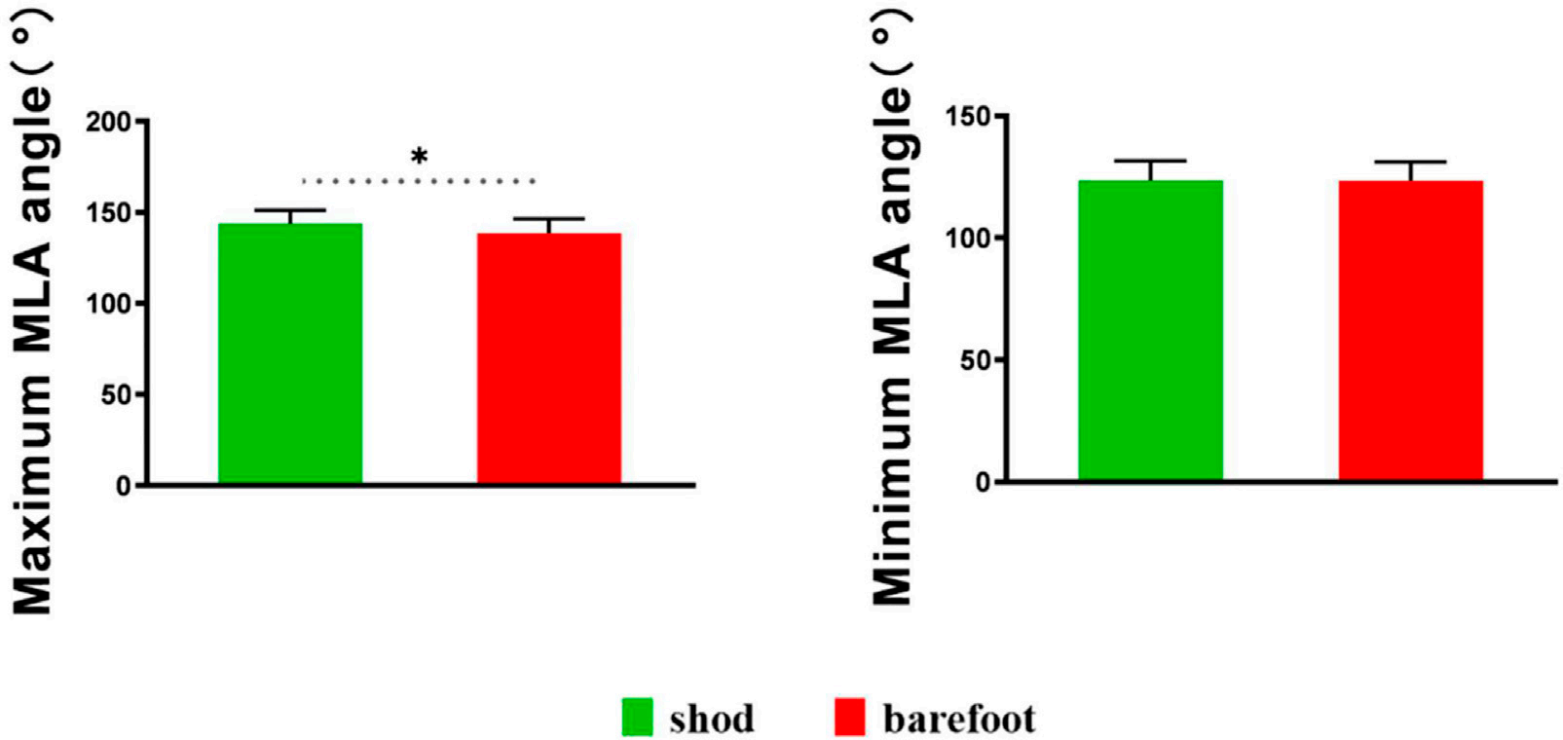
Maximum and minimum MLA angles in shod and barefoot conditions. *: significant differences existed between the shod and barefoot conditions, *p* < 0.05.

Compared with barefoot, the MLA angles were significantly smaller at initial contact (*p* = 0.004), 10% (*p* < 0.001), 20% (*p* < 0.001), 30% (*p* = 0.001), 40% (*p* = 0.001), 50% (*p* = 0.001), 60% (*p* = 0.016), and 70% (*p* = 0.043) of the stance phase in the shod condition (Figure 8). The MLA angle at the initial contact (*p* = 0.004), maximum angle (*p* = 0.002), and ROM (*p* < 0.001) in the shod condition were significantly smaller, whereas no significant difference was observed between the shod and barefoot conditions at the minimum angle (Figure 9) (Table 2).

**TABLE 2:** Comparison of MLA angle at initial contact and ROM of MLA angle in shod and barefoot conditions.

	Condition	MLA angle (°)
Initial contact	barefoot	129.62 ± 8.43*
	shod	126.13 ± 7.67*
ROM	barefoot	20.32 ± 4.20*
	shod	15.01 ± 3.98*

*: compared with barefoot, significant differences existed in the shod condition, *p* < 0.05.

## 4 Discussion

The study showed that compared with the barefoot, the medial, anterior, superior translation, and IR, DF angles of the first metatarsal relative to the calcaneus were reduced in the shod condition at early and mid-stances. The study also revealed that compared with barefoot, the MLA angles at the initial contact, maximal MLA angle, ROM of MLA angles, and MLA angles from early to mid-stances were reduced. These results were consistent with the first hypothesis, which states that shoe wearing restricts partial 6DOF movement of the first metatarsal relative to the calcaneus. Inconsistent with the study hypothesis, no significant differences were observed in the minimal MLA angles between shod and barefoot conditions.

Compared with the barefoot condition, the maximal MLA angle and the ROM of MLA angles were significantly reduced in the shod condition, which partly coincide with the results of previous studies (Kelly et al., 2016; Holowka et al., 2021). Kelly et al. (2016) reported that running with shoes led to a reduction in the magnitude of MLA compression and recoil. Holowka et al. (2021) discovered that compared with barefoot, running with viscose-elastic midsole shoes reduced the ROM of MLA angles. These results based on skin-marker motion capture were consistent with the findings of this study, that is, shoes restricted the MLA movement in the sagittal plane, indicating that shoe wearing limited the compression and recoil of the MLA. The MLA may be affected by the compressive stiffness of footwear, and footwear compressive stiffness and MLA stiffness are related through an in-series spring system, such as the leg–surface relationship (Kelly et al., 2016). The in-series spring system is believed to maintain the constant overall stiffness; more compliant footwear should cause individuals to increase their MLA stiffness during running (Butler et al., 2003). Compared with wearing shoes that are more compliant, barefoot running is in direct contact with the stiff ground. Therefore, to maintain the constant overall stiffness, the human body increased the MLA stiffness under shod conditions and achieved the adjustment by decreasing the compression of MLA. MLA stiffness is defined as the ability to resist compression during loading and is commonly associated with arch height (Noro et al., 2021). Both extremes of MLA height have been associated with an increased risk of foot injuries, with both overly-stiff and overly-compliant arched thought to be poor shock absorbers (Simkin et al., 1989). Additionally, high-arched individuals with larger MLA stiffness were more likely to develop tibial and femoral stress fractures (Williams et al., 2001). The compression and recoil of MLA are related to the storage and release of elastic energy during running. Ker et al. (1987) identified the MLA as an elastic storage-return mechanism and estimated that approximately 17% of the mechanical work of running can be stored and returned through the compression and recoil of the MLA over the stance phase. Accordingly, we hypothesized that the reduced compression and recoil of the MLA may influence the spring-like function of MLA and limit the stored and released elastic energy.

This study, based on high-speed DFIS, observed the movement of the first metatarsal with respect to the calcaneus during running under barefoot and shod conditions. This study showed no significant differences in the peak lateral/medial, anterior/posterior, and inferior/superior translation and PF/DF, AB/AD, and IR/ER in the movement of the first metatarsal relative to the calcaneus between barefoot and shod conditions. However, in the early and mid-stances, the metatarsal anterosuperior was smaller in the shod condition compared with the barefoot. Noh et al. (2015) believed that given the decreased displacement of the metatarsal, muscle activation of the plantar flexors, such as flexor digitorum brevis muscle (FDB), which insert onto the bone, was increased. In line with the results, Kelly et al. (2016) observed that compared with barefoot, the compression and recoil of the MLA reduced, and peak FDB activation was greater when running with shoes. According to previous studies, the magnitude of MLA compression decreased accompanied by an increased muscle activation due to the replacement of the spring-like function of the MLA with muscle work, which may increase the metabolic cost (Alexander, 1991; Stearne et al., 2016; Cigoja et al., 2021). Stearne et al. (2016) observed that limiting the MLA compression by the use of custom insoles, which restricted arch compression to 20 and 40%, resulted in increases of 1 and 2.5% in metabolic energy cost, respectively. Perl et al. (2012) also revealed a significant 3% increase in metabolic costs for traditional shoe wearing compared with minimalist shoes that were similar to barefoot running. Although metabolic costs and muscle activation were not directly measured in this study, combining the results of this and previous research, we agreed with the opinion that shoe wearing may increase metabolic costs compared with barefoot running.

It has been shown that wearing minimal shoes (used to mimic barefoot running) is associated with increases in intrinsic foot muscle size and MLA height (Lieberman et al., 2010; Lieberman, 2012). Miller et al. (2014) and Johnson et al. (2016) randomly assigned participants to wear minimal shoes or traditional shoes for weeks of running, and both found that participants in the minimal shoes group were significantly increased in cross-sectional area (CSA) of abductor hallucis muscles than in the traditional shoes group. Miller et al. (2014) also found that CSA of abductor digiti minimi muscles was larger and MLA height was higher in the minimal shoes group. Similar to these studies, Chen et al. (2016) found that overall foot muscle volume was larger in the minimal shoes group but not in the traditional shoes group. These studies suggested that wearing minimal footwear for the long term can help increase foot strength and MLA height. Conversely, shoe-wearing may be associated with weaker intrinsic foot muscles that may potentially lower MLA, further collapsing the foot.

Based on high-speed DFIS, this study revealed that compared with barefoot, MLA angle at the initial contact and maximal MLA angle were significantly smaller in the shod condition. However, Kelly et al. (2016) reported differences in the maximal and minimal angles between shod and barefoot running; however, the MLA angle at initial contact was similar for both conditions. We hypothesized that the differences between this study and those of previous research were mainly due to the different methods of measuring the MLA angle. Previous studies on the measurement of MLA angle were mostly based on infrared motion-capture techniques and referred to the Rizzoli foot model, which defines the MLA angle as the 2D angle between the projections of two vectors on the sagittal plane of the foot, thus simulating X-ray image-based clinical measurements of the MLA (Leardini et al., 2007; Portinaro et al., 2014). In this study, we defined the MLA angle as the angle between two 3D vectors. Caravaggi et al. (2021) observed that during the dynamic task, the variability of the 3D angle was consistently lower than the 2D angle, which was projected to the sagittal plane, suggesting that the 3D angle reflected the deformation of the MLA more accurately. Therefore, this study, based on high-speed DFIS, may observe the movement of the MLA more accurately.

This study encountered several limitations. Only male runners were recruited, and gender differences were not explored. All participants were habitual shoe-wearing runners, and when immediately transitioning from shod to barefoot running, this transition may lead to a decreased local dynamic stability (Ekizos et al., 2017). In addition, future studies should further explore the effect of training on the *in vivo* kinematics of the MLA during running based on high-speed DFIS.

## 5 Conclusion

The study investigated the MLA angle and the 6DOF movement of the first metatarsal relative to the calcaneus in the whole stance phase, which provided valuable insights into the MLA kinematic under barefoot and shod conditions during running and lend more accurate information of MLA in the whole stance phase. The results showed that shoe wearing restricted partial 6DOF movement of the MLA, especially reducing the magnitude of MLA compression and recoil, suggesting that shoes limited the spring-like function of the MLA. The restricted spring-like function of the MLA may affect the storage and release of elastic energy during running.

## Data Availability

The original contributions presented in the study are included in the article/supplementary material, further inquiries can be directed to the corresponding authors.
